# Prognosis of Syncope With Head Injury: a Tertiary Center Perspective

**DOI:** 10.3389/fcvm.2020.00125

**Published:** 2020-07-23

**Authors:** Stanisław Furtan, Paweł Pochciał, Dariusz Timler, Fabrizio Ricci, Richard Sutton, Artur Fedorowski, Dorota Zyśko

**Affiliations:** ^1^Department of Emergency Medicine, Wrocław Medical University, Wroclaw, Poland; ^2^Department of Emergency Medicine and Disaster Medicine, Medical University of Lodz, Łódz, Poland; ^3^Department of Neuroscience, Imaging and Clinical Sciences, Institute for Advanced Biomedical Technologies, G. D'Annunzio University, Chieti, Italy; ^4^Department of Clinical Sciences, Faculty of Medicine, Clinical Research Center, Lund University, Malmö, Sweden; ^5^National Heart and Lung Institute, Imperial College, Hammersmith Hospital Campus, London, United Kingdom; ^6^Department of Cardiology, Skåne University Hospital, Malmö, Sweden

**Keywords:** syncope, head injury, mortality, prognosis, Glasgow Coma Scale

## Abstract

**Aim:** Head injury is the most common trauma occurring in syncope. We aimed to assess whether syncope as cause of head-trauma affects short-and long-term prognosis.

**Methods:** From a database retrospective analysis of 97,014 individuals attending Emergency Department (ED), we selected data of patients with traumatic head injury including age, gender, injury mechanism, brain imaging, multiple traumas, bone fracture, intracranial bleeding, and mortality. Mean follow-up was 6.4 ± 1.8 years. Outcome data were obtained from a digital national population register. The study population included 3,470 ED head injury patients: 117 of them (50.0 ± 23.6 years, 42.7% men) reported syncope as cause of head trauma and 3,315 (32.2 ± 21.1 years, 68.5% men) without syncope preceding head trauma.

**Results:** Thirty-day mortality was low and similar in traumatic head injury with or without syncope. One year and long-term all-cause mortality were both significantly higher in syncopal vs. non-syncopal traumatic head injury (11.1 vs. 2.8% and 32 vs. 10.2%, respectively; both *p* < 0.001). In adjusted logistic regression analysis, death between 121st-day and 1 year in patients with head-trauma was associated with male gender [odds ratio (OR): 6.48; 95% CI: 2.59–16.25], advancing age (per year) (OR 1.09; 95% CI 1.07–1.11), Glasgow Coma Scale < 13 (OR: 6.18; 95% CI:1.68–22.8), bone fracture (OR 4.72; 95% CI 2.13–10.5), and syncope (OR 3.70; 95% CI: 1;48–9.31). In multivariable Cox regression analysis, syncope was one of the strongest independent predictors of long-term all-cause death (hazard ratio: 1.95; 95% CI 1.37–2.78).

**Conclusion:** In patients with head trauma, history of syncope preceding injury does not increase 30-day all-cause mortality but portends increased 1 year and long-term mortality.

## Introduction

Injuries are the third commonest cause of death worldwide following circulatory diseases and cancer. About half of deaths due to injuries occur as a result of head trauma (1, 2). Loss of consciousness due to injury is a strong recommendation for head computer tomography (CT) scan (2). However, in patients presenting to ED with both syncope and head trauma, it is often difficult to decide whether head trauma was preceded by syncope or whether transient loss of consciousness (TLOC) was due to brain injury (3).

Most injuries that occur as a result of syncope are head injuries (4, 5). Falls as a result of syncope may vary in their severity; cardiac syncope results generally in a more rapid decrease in supply of blood to the brain than vasovagal syncope and may be associated with worse prognosis due to both the underlying cardiac mechanism of syncope and the severity of head injury, as it stems from the “dead weight” of an unprotected fall (5, 6). A fall due to syncope is usually from the patient's height, however, falls from height (steps, roof) are also possible. Moreover, high-energy injury may rarely be caused by driving accidents due to syncope (7, 8).

Traumatic injuries occur in 15–45% of syncopal patients, and severe injuries in 0.6–4.8% depending on the studied population (4, 9–15). Patients with traumatic injuries due to syncope have shown a higher 1 year mortality than other patients with syncope (11, 12). Less is known of the importance of syncope in the prognosis of the patients with traumatic head injury. The long-term prognosis in head trauma patients may be related not only to the severity of head injury but also to the underlying condition, which predisposes to trauma such as alcoholism, drug addiction, or concomitant chronic diseases (16–20). The aim of this study was to assess whether syncope as a cause of head trauma affects short- and long-term prognosis in patients presenting with head injury.

## Methods

We performed a retrospective analysis of medical records of 97,014 patients admitted during two periods—from January 2007 to June 2008 and from January 2009 to June 2010—to an emergency department (ED) in Zgorzelec, Poland, a regional trauma center with a catchment area of ~200,000 citizens. We identified patients admitted because of traumatic head injury and retrieved the following data: age, gender, mechanism of injury (high or low energy), presence of multiple trauma, head computer tomography (CT), bone fracture, intracranial bleeding, and specialist consultations. Thirty-day, 1 year and survival at longest follow-up available were assessed using the data obtained from a Polish national population register (PESEL), including the date of death, if applicable. The study was approved by the Bioethical Commission of Wroclaw Medical University, Poland (No. 552/2012).

## Statistical Analysis

Continuous variables were presented as mean and standard deviation and compared using Student's *t*-test, whereas discrete variables were presented as numbers and percentages, and compared using Pearson's ch^2^-test. Logistic regression analysis was performed to identify factors associated with 1-month and 1 year survival, for the latter, after excluding patients who had died within 120-days of the index head injury. Further, logistic regression analysis was performed to identify factors related to intracranial hemorrhage. Rates of overall survival were estimated by means of the Kaplan–Meier method and were compared between head-trauma with and without syncope by log-rank test. A Cox proportional hazards analysis was used to calculate the adjusted hazard ratio of all-cause mortality by each variable. All recorded variables were forced to enter into the final model. We performed Schoenfeld's test to check the validity of proportional hazards assumptions and checked the validity of constant incidence ratios over the follow-up using Nelson-Aalen's cumulative hazard estimates. *P* < 0.05 was considered as significant. The statistical analysis was performed using IBM SPSS Statistics version 25.0 software (SPSS Inc. Chicago, Illinois, USA).

## Results

The study group consisted of 3,470 patients admitted to the ED because of head injury (3.6% of total patient population). Of those, 117 patients had reported that head trauma was preceded by a sudden loss of consciousness and muscle tone, and were classified as head trauma due to syncope. Thirty-eight patients (1%) were excluded due to incomplete or incorrect data, yielding the final study sample of 3,432 patients. Mean follow-up was 6.4 ± 1.8 years.

The main characteristics of syncopal (*n* = 117) and non-syncopal head-trauma patients (*n* = 3,315) are presented in Table 1. The syncopal head-trauma patients were older, more likely women, had more frequently undergone brain CT, were more likely to receive specialist consultations and, also, more likely to be admitted to hospital than those with non-syncope related head-injury.

**Table 1:** Baseline demographics and clinical data of emergency department patients with and without syncope as the cause of head-trauma.

**Covariates**	**Syncope + *n* = 117**	**Syncope—*n* = 3,315**	***P*-value**
Age, years	50.0+/−23.6	32.2+/−21.1	0.001
Male gender, *n* (%)	50 (42.7)	2,266 (68.5)	0.001
30-day mortality, *n* (%)	2 (1.7)	31 (0.9)	0.399
120-day mortality, *n* (%)	6 (5.1)	60 (1.8)	0.010
1 year mortality, *n* (%)	13 (11.1)	92 (2.8)	0.001
High energy mechanism of injury, *n* (%)	10 (8.5)	1.355 (40.4)	0.001
Alcohol abuse, *n* (%)	13 (11.1)	472 (14.5)	0.294
Bone fracture, *n* (%)	7 (6.0)	285 (8.6)	0.316
ICH at CT scan, *n* (%)	3 (2,6)	20 (0.8)	0.151
GCS <13, *n* (%)	3 (2.6)	60 (2.1)	0.761
Unconsciousness immediately after head trauma, *n* (%)	117 (100.0)	406 (12.3)	0.001
Head CT, *n* (%)	48 (41.0)	480 (15.5)	0.001
Neurology consultation, *n* (%)	62 (53.0)	613 (18.5)	0.001
Neurosurgery consultation, *n* (%)	3 (2.6)	29 (0.9)	0.067
Cardiology consultation, *n* (%)	5 (4.3)	3 (0.1)	0.001
Hospital admission, *n* (%)	42 (35.9)	634 (19.2)	0.001

*CT, computed tomography; GCS, Glasgow Coma Scale; ICH, intracranial hemorrhage*.

Thirty-day mortality in patients without syncope not admitted to hospital, with syncope not admitted to hospital, without syncope admitted to hospital, and with syncope admitted to hospital was, respectively, 0.4, 0.0, 3.3, and 4.8% (Table 2).

**Table 2:** Baseline characteristics of patients who died within 30-days and survivors at 30-days.

**Covariates**	**Death within 30-days *n* = 33**	**Survivors at 30-days *n* = 3,399**	***p*-value**
Age, years	54.5 ± 22.1	32.6 ± 21.3	<0.001
Male gender, *n* (%)	26 (78.8)	2,290 (67.4)	0.17
GCS < 13, *n* (%)	19 (57.6)	52 (1.5)	<0.001
High-energy trauma, *n* (%)	20 (60.6)	1,332 (39.2)	0.007
Syncope, *n* (%)	2 (6.1)	115 (3.4)	0.40
Epilepsy, *n* (%)	2 (6.1)	62 (1.8)	0.07
Alcohol abuse, *n* (%)	5 (15.2)	480 (14.1)	0.87
Bone fracture, *n* (%)	4 (12.1)	288 (8.5)	0.45
ICH, *n* (%)	9 (27.3)	22 (0.7)	<0.001

*GCS, Glasgow Coma Scale; ICH, intracranial hemorrhage*.

One year mortality in patients without syncope who were not hospitalized was 1.9% whereas among those who were hospitalized was 6.7%. One year mortality in patients with syncope who were not hospitalized was 6.7% whereas among those who were hospitalized was 19.0% (Table 3).

**Table 3:** Baseline characteristics of patients who survived longer than 1 year and those who died between the 121st day after injury and 1 year.

**Covariates**	**Survivors > 1 year *n* = 66**	**Survivors ≤ 1 year *n* = 3,366**	***P*-value**
Age, years	64,359	31,899	<0.001
Male gender, *n* (%)	32 (82.1)	2,240 (67.4)	0.052
Syncope, *n* (%)	7 (18.0)	104 (3.1)	<0.001
Epilepsy, *n* (%)	1 (2.6)	57 (1.7)	0.83
Alcohol abuse, *n* (%)	11 (28.2)	462 (13.9)	<0.001
Bone fracture, *n* (%)	10 (25.6)	273 (8.2)	<0.001
ICH, *n* (%)	1 (2.6)	19 (0.6)	0.57
Polytrauma, *n* (%)	3 (7.7)	111 (3.3)	0.29
High energy traumatic injury, *n* (%)	14 (35.9)	1,310 (39.4)	0.78
GCS < 13, *n* (%)	3 (7.7)	75 (1.4)	0.008

*GCS, Glasgow Coma Scale; ICH, intracranial hemorrhage*.

In all head-trauma patients, 30-day mortality was related to older age (OR per year 1.05; 95% CI 1.03–1.07, *p* = 0.01), Glasgow Coma Scale (GCS) <13 (OR: 76.1; 95% CI 34.8–166.5 *p* < 0.001) presence of intracranial hemorrhage (OR 2.98; 95% CI 1.11–7.98, *p* = 0.03). Syncope preceding head injury was not predictive of short-term mortality.

Logistic regression analysis revealed that death between 121st and 365th days was predicted by male gender (OR: 6.48; 95% CI: 2.59–16.25 *p* < 0.001), older age (per year) (OR: 1.09; 95% CI: 1.07–1.11 *p* < 0.001), GCS < 13 at admission (OR: 6.18; 95% CI: 1.68–22.8 *p* < 0.001), syncope as a cause of trauma (OR:3.70; 95% CI: 1.48–9.31, *p* < 0.001), and bone fracture (OR:4.72; 95% CI: 2.13–10.52, *p* < 0.001). Logistic regression analysis showed that increased risk for intracranial bleeding was related to male sex (OR 5.4; 95% CI 1.6–17.8, *p* < 0.006), older age (OR per year 1.022; 95% CI 1.003–1.042, *p* = 0.03), and GCS < 13 (OR 99.0; 95% CI: 44.0–222.9, *p* < 0.001) at admission. Syncope was a borderline factor (OR 4.0; 95% CI: 0.92–17.3, *p* = 0.06).

Patients who died within 30-days were older and were more likely to have a GCS < 13 at admission, high-energy injury, and intracranial hemorrhage found on CT scan.

**Figure 1:**
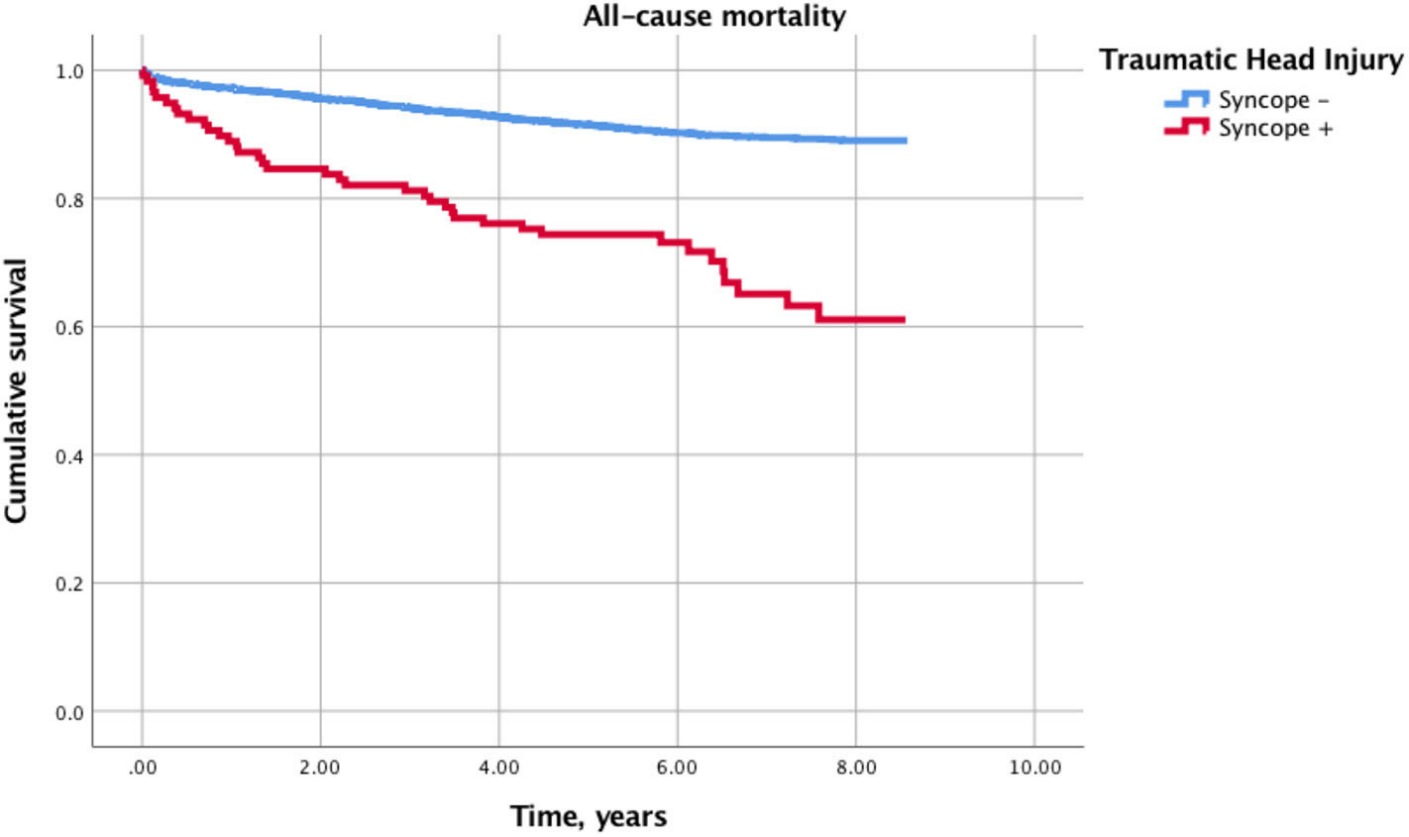
Kaplan-Meier curves for cumulative incidence of all-cause death in syncopal vs. non-syncopal traumatic head injury.

As shown by the Kaplan-Meier estimate, syncope-related head injuries were associated with significantly higher all-cause mortality compared with non-syncopal head injuries (Log-rank (Mantel-Cox): *p* < 0.001) (Figure 1). Furthermore, in multivariable Cox regression analysis, syncope, epilepsy, GCS < 13 and alcohol abuse were the strongest independent predictors of long-term all-cause mortality (Table 4).

**Table 4:** Predictors of long-term all-cause mortality after head injury in a multivariable-adjusted Cox regression model.

**Covariates**	**Hazard ratio**	**95% CI**	***P*-value**
Age, years	1.070	1.074–10.76	<0.001
Male gender, *n* (%)	1.573	1.232–2.009	<0.001
Syncope, *n* (%)	1.949	1.366–2.780	<0.001
Epilepsy, *n* (%)	2.039	1.156–3.597	0.014
Alcohol abuse, *n* (%)	1.929	1.508–2.467	<0.001
Bone fracture, *n* (%)	1.397	1.009–1.934	0.044
High-energy traumatic injury, *n* (%)	1.301	1.036–1.635	0.024
GCS < 13, *n* (%)	4.655	3.047–7.112	<0.001

*GCS, Glasgow Coma Scale*.

## Discussion

In this study, we have observed that syncope precipitating head injury does not increase short-term mortality but is associated with increased longer-term mortality. The study also showed the rather more expected result that 30-day mortality in Emergency Department patients admitted after head injury is associated with older age, intracranial bleeding, and Glasgow coma scale <13 at admission concordant with other similar studies (21).

Syncope as a cause of injury is not a factor typically associated with 30-day mortality in both single and multivariate analyses. These results indicate the significance of damage to the central nervous system in assessment of short-term prognosis. In patients with loss of consciousness the interview is difficult, if at all possible, rendering the identification of fainting as a cause of head injury often underestimated. In the present study of patients with syncope followed by head injury, the 30-day mortality rate was 1.7%. The short-term mortality of patients with syncope in other similar studies was also low (12). The presence of an injury is not a factor influencing the prognosis in patients after fainting. The risk of death during short-term observation is related not to the loss of consciousness itself, but to the illness that underlies syncope.

In STePS, 5 patients died from pulmonary edema, aortic dissection, pulmonary embolism and stroke, similarly 10 patients died in the EGSYS study, which was 1% of the study group (9, 12). The cause of death in this group was sudden cardiac in one patient, 2 patients had lung disease, advanced cancer in 3 patients and undetermined cause in 1 patient. Long-term prognosis was also associated with age, presence of cancer, cerebrovascular disease, structural heart disease, and ventricular arrhythmia (12).

In the analysis of 1 year survival in the subgroup of patients who achieved 4-month survival, the risk factors for death were age, male gender, syncope, bone fracture, decreased GCS scoring, and older age. High-energy injury was not an independent risk factor for death in this group of patients, however, the fracture that accompanied the trauma still remained a risk factor for death. Bone fracture that accompanies a head injury can be a sign of a significant force of injury. In the case of syncope, the occurrence of an injury is more frequent in cardiac syncope, in which a sudden “dead weight” fall occurs compared with reflex syncope where the decrease in blood pressure is usually slower resulting in a crumpling fall, usually accompanied by less injuries. Hino et al. reported that patients with loss of consciousness and maxillofacial fractures tend to have more severe maxillofacial injuries than those without loss of consciousness (22). The absence of links between bone fracture and long-term prognosis (after exclusion from analysis of patients who died within 4 months) indicates that bone fracture should be considered a surrogate of other factors related to long-term survival and not directly determining survival. These other factors may include not only the occurrence of cardiac syncope, but also the patient's lifestyle, increasing the risk of further injuries. Likewise, patients with traumatic injuries due to syncope who die during long-term observation have sustained more severe injury (23).

One of the important findings of this study is that syncope causing head trauma predicts mortality in patients who survive the first 4 months. Annual mortality in the whole group of patients admitted to ED after head injury who survive the first 4 months is 1% and is similar to the annual mortality of patients admitted to ED due to eye and ear diseases (24). In the subgroup of patients with syncope, mortality is over 6%. Syncope in this group in both single and multivariate analyses is an independent risk factor for death.

Mortality in a patient after head injury may depend on the direct effects of head injury, distant consequences of head injury, and coexisting conditions leading to syncope and injury. The assessment of each of these three seemingly obvious components can actually be very difficult.

The direct effects of a head injury can be assessed using the level of consciousness according to the GCS when admitted to the ED. However, it should be remembered that disturbance of consciousness is not always a direct consequence of brain injury but may be associated with consciousness disorders due to alcohol or psychoactive substance toxicity, postictal state following epileptic seizure or prolonged arrhythmia leading to central hypoxia (2, 25). Some of these factors may affect survival regardless of the injury. In the present study, the influence of low admission GCS score on the annual survival was still present after exclusion of patients who died within the first 4 months of injury. Moreover, the risk of fall-related injuries is increased among patients hospitalized for unexplained syncope, regardless of syncope mechanism (26).

Among mechanisms of head injury, we traditionally distinguish between high- and low-energy impacts. It is expected that greater impacts will lead to greater head damage. However, this dependency is not directly proportional. The direct effects of head injury depend not only on the force itself but also on the anatomical structure of the head and which parts are affected, concurrent anticoagulant therapy and the possibility of self-defense against the trauma. The relationship between syncope and injury may be further complicated by the fact that as a result of fainting or injury, the patient may have retrograde amnesia, which may lead to confusion regarding the facts relating to fainting and the injury scenario (27). Moreover, presence of manifest cardiovascular disease is a risk factor which increases, by a factor of 5–10, the mortality risk in patients with trauma (28, 29), and syncope may be a direct consequence of an acute circulatory disturbance caused by cardiovascular disease, undetected at ED presentation.

## Limitations

There are some important study imitations that should be mentioned. The first of these is the retrospective nature of the study and inextricably related factors such as incomplete documentation. Second, the data regarding concomitant medications was not available in the available medical records. Third, due to the retrospective nature of the study, the ideal syncope history was not necessarily complete with only that recorded in the patient's file being available. Fourth, the study has been conducted only in one Eastern European country which may limit its generalizability. However, access to the digital national data on total mortality and the large group of analyzed patients increase the validity of our study findings.

## Conclusions

1. Syncope-related head injury does not increase short-term mortality, but is associated with increased 1 year and long-term mortality compared with all patients admitted to the Emergency Department with head injuries.

2. Patients with syncope as the cause of head injury more frequently suffer low energy traumatic injury; CT scans and hospital admissions are both increased in this patient population compared with those who present head injury without preceding syncope.

3. Intracranial hemorrhage occurs slightly more often in patients after syncope, but syncope is not an independent risk factor for intracranial hemorrhage. However, older age, male gender, and lower Glasgow Coma Scale score on admission are important risk factors.

## Data Availability Statement

The datasets generated for this study are available on request to the corresponding author.

## Ethics Statement

The studies involving human participants were reviewed and approved by Bioethical Commission of Wroclaw Medical University, Poland (Grant No. 552/2012). The patients/participants provided their written informed consent to participate in this study.

## Author Contributions

SF, PP, DT, and DZ designed the study. SF, PP, and DZ collected the data. DZ, AF, and FR performed the statistical analyses. SF, DT, FR, RS, AF, and DZ searched the literature. SF, PP, DT, and DZ drafted the manuscript. FR, RS, and AF reviewed and corrected the manuscript. All authors take full responsibility for the contents of the manuscript.

## Conflict of Interest

The authors declare that the research was conducted in the absence of any commercial or financial relationships that could be construed as a potential conflict of interest.
